# Pigmented squamous cell carcinoma *in situ* of the nail: An important differential diagnosis of melanonychia striata—Evaluation by dermatoscopy and *in vivo* confocal microscopy

**DOI:** 10.1016/j.jdcr.2024.12.035

**Published:** 2025-01-24

**Authors:** Juliana Areas de Souza Lima B. Ferreira, Juliana Casagrande Tavoloni Braga, Eduardo Bertolli, Clovis Antonio Lopes Pinto

**Affiliations:** a Faculdade de Medicina de Jundiaí (FMJ), Jundiai, São Paulo, Brazil; b A.C. Camargo Cancer Center, São Paulo, São Paulo, Brazil

**Keywords:** confocal microscopy, dermatoscopy, nail surgery, pigmented Bowen disease

## Introduction

Squamous cell carcinoma (SCC) of the nail is a malignant tumor that usually originates below the nail plate and grows slowly.^1^^,^^2^ Clinical manifestations depend on the site of involvement in the nail unit and include the following: onycholysis, hyperkeratosis, erosion, erythronychia, and melanonychia striata (resulting from melanocytic activation by the tumor).^2^ Different clinical presentations may coexist in nail SCC, resembling benign or malignant lesions.^2^ For this reason, there is often a delay in diagnosis. Although a thorough dermatological examination and the use of diagnostic tools such as dermatoscopy help in the diagnostic hypothesis of this tumor,^2^ an accurate diagnosis is made by performing a biopsy followed with histopathologic evaluation,^3^ but biopsy is a painful procedure that can cause permanent nail dystrophy. In addition, choosing the site for the biopsy can be a challenge even with the aid of intraoperative dermatoscopy.^4^ To minimize these problems and improve diagnostic accuracy, new imaging techniques that complement more widely used preliminary noninvasive testing or evaluation of these suspicious lesions can be used, such as confocal microscopy.

## Case report

**Fig 1:**
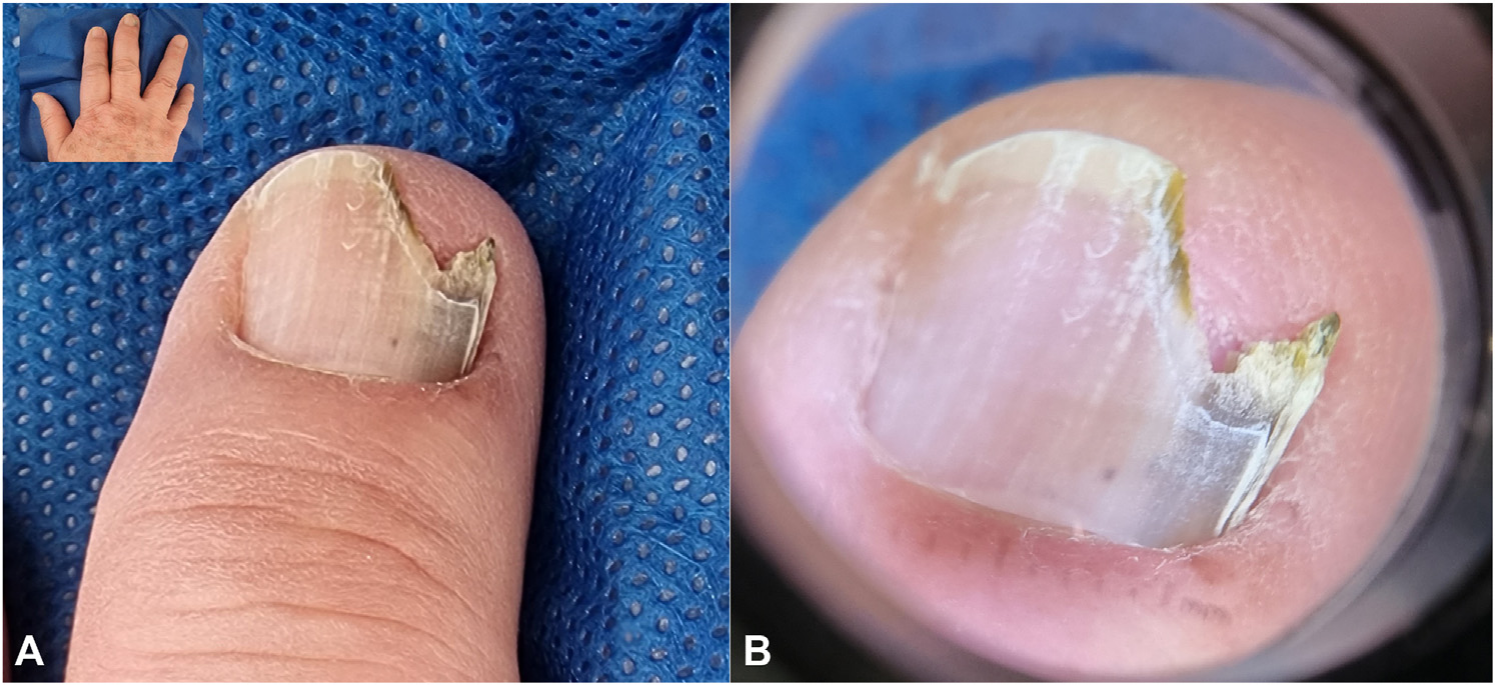
**A,** Clinical image and detail of the nail plate of the third right long finger showing onycholysis with hyperkeratosis in addition to a 3 mm brown longitudinal pigmentation. **B,** Dermatoscopic image of the nail plate (onychoscopy) showing onycholysis and distal hyperkeratosis of the nail plate in addition to a 3 mm brown striated melanonychia, with irregular brown parallel longitudinal lines and a break in parallelism.

**Fig 2:**
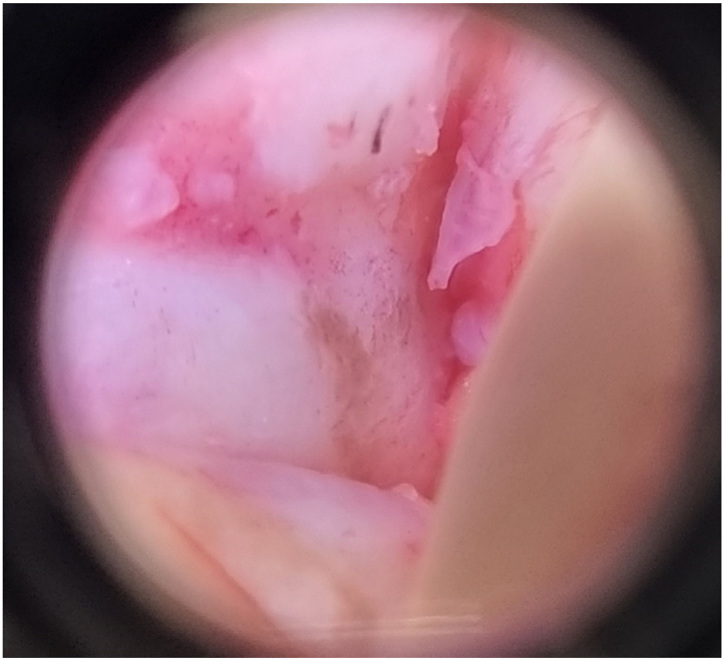
Intraoperative dermatoscopic image: irregular brown pigmentation with irregular striped pattern.

**Fig 3:**
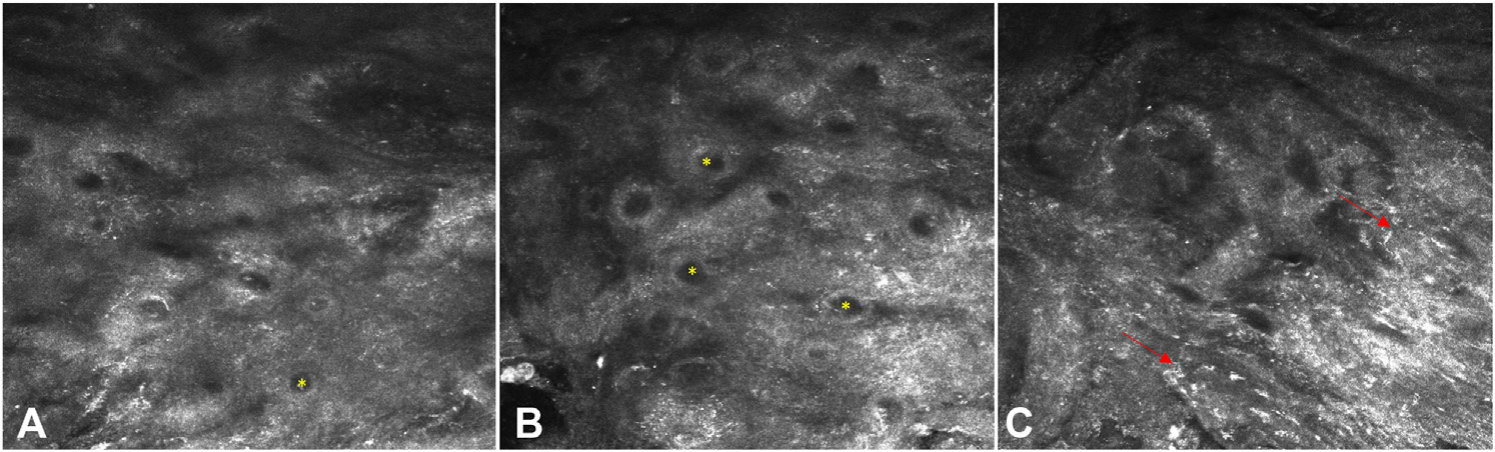
Individual confocal microscopy images taken intraoperatively. Panels (**A, B**) showing small, irregular papillae (*yellow asterisk*) with widening of the interpapillary space at the dermoepidermal junction (DEJ). **C,** DEJ with small, irregular papillae and presence of dendritic cells (*red arrows*).

**Fig 4:**
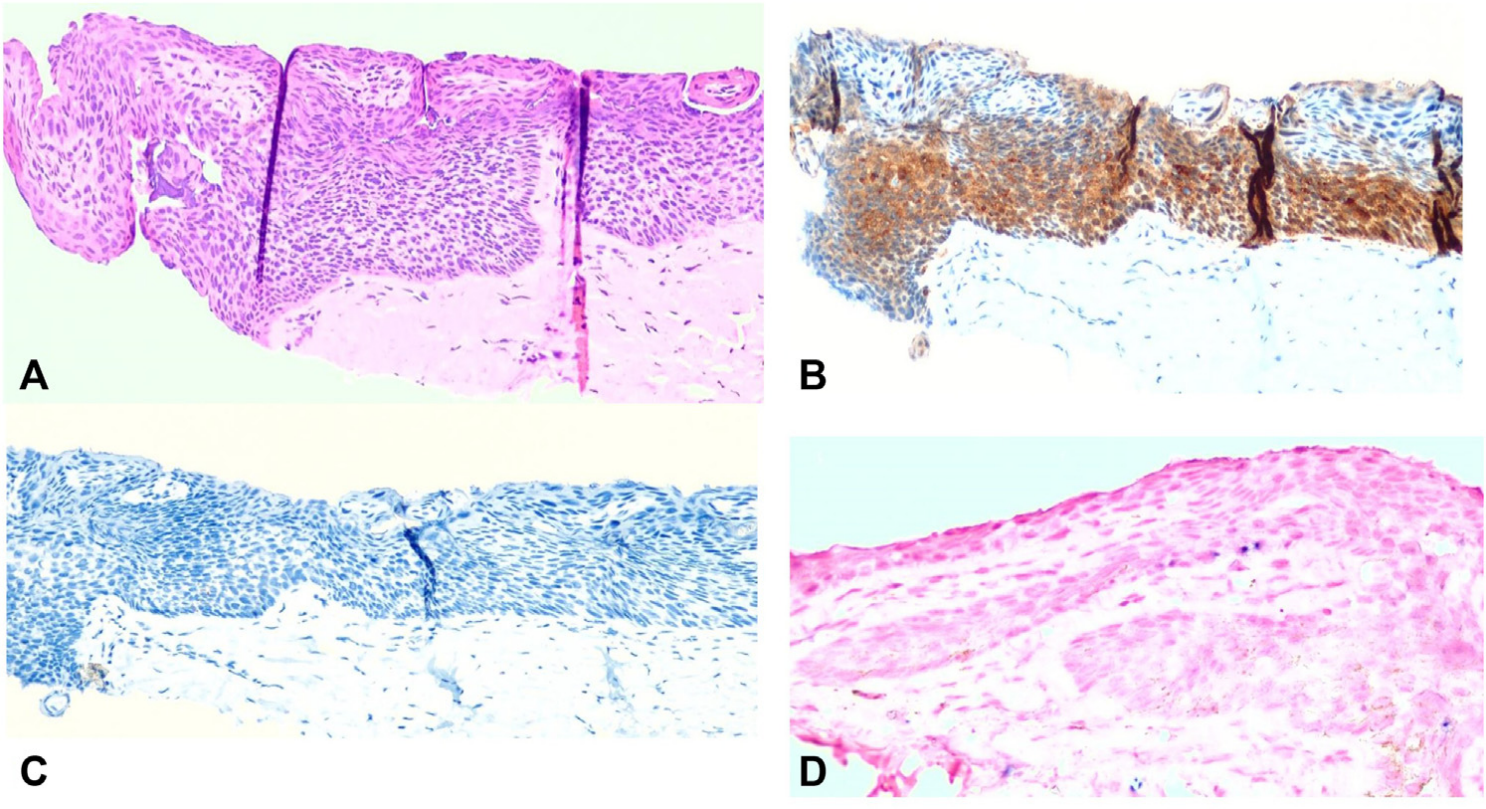
**A,** Nail bed with slight hyperpigmentation of the basal layer and atypia of the epithelium. **B,** Positive p16 immunohistochemical study. **C,** Negative SOX 10 immunohistochemical study for melanocytes. **D,** Chromogenic *in situ* hybridization for high-risk human papillomavirus subtypes (*blue dots*: presence of viral DNA). (**A,** Hematoxylin-eosin stain; original magnifications: **A-C,** ×200; **D,** ×400.)

An 80-year-old man with Fitzpatrick skin type III sought a dermatologist reporting pigmentation in the nail plate of the third right long finger, for 7 months with progressive growth. The dermatological examination showed onycholysis with mild distal hyperkeratosis and brown pigmentation (Fig 1). Dermatoscopy of the nail plate (onychoscopy) showed onycholysis and distal hyperkeratosis and 3 mm striated melanonychia, with irregular parallel brown longitudinal lines in their spaces, thickness and coloration and with parallel breaks without bleeding (Fig 1). Therefore, a biopsy of the nail bed was indicated. After local anesthesia of the third right long finger, the nail plate was avulsed, exposing the bed and matrix and subsequent evaluation with polarized dermatoscopy followed by *in vivo* confocal microscopy intraoperatively to define the site of the incisional biopsy. Intraoperative dermatoscopy showed mild brown pigmentation with a striped pattern (Fig 2). Confocal microscopy showed an area with cellular atypia in all layers of the epidermis, dendritic cells and small, irregular papillae, and widening of the interpapillary space at the dermoepidermal junction (Fig 3), which guided the site to perform the shaving biopsy of the nail bed. Tissue histopathology using hematoxylin-eosin staining revealed mild hyperpigmentation of the basal layer and epithelial atypia. Immunohistochemical studies revealed positive p16 and negative SOX 10, which helped confirm a diagnosis of SCC *in situ*. *In situ* hybridization was performed to investigate high-risk human papillomavirus infection, which was positive (Fig 4). The patient underwent conservative surgery with removal of the nail apparatus, and the histopathologic examination confirmed residual SCC *in situ* with free margins.

## Discussion

SCC of the nail unit is a rare but often underappreciated neoplasm.^5^ The estimated incidence for SCC of the nail unit has ranged from 3 cases in 250,000 hospital admissions to 14 cases in 50,000 dermatologic consultations.^5^ The sex ratio between men and women is 2:1, with a peak incidence between 50 and 69 years of age.^6^ It especially affects the first thumb finger, with the second index, and third long fingers of the dominant hand being other common locations.^7^ Lymph node involvement by SCC, although a rare event, can occur. Factors that favor the development of nail SCC are exposure to ionizing radiation, high-risk human papillomavirus, especially type 16 (in 74% of cases), and chronic trauma.^1^^,^^2^

Melanonychia striata is usually the most common clinical manifestation of melanocytic lesions, but other diagnoses such as hematomas, infections, and keratinocytic tumors cannot be ruled out.^4^^,^^8^ Onychoscopy helps in the evaluation of nail diseases^2^ and the findings commonly found in SCC are onycholysis, irregular vascularization with a rough surface, although none of these signs are exclusive to the tumor.^2^^,^^6^ A recently used method to investigate nail tumors is confocal microscopy.^2^ Debarbieux et al^9^ described an excellent correlation of malignant epithelial tumors with the observation of marked cytological atypia, such as nuclear pleomorphism and densely packed and irregularly organized nuclei, typical of SCC in *ex vivo* confocal microscopy, suggesting that this technique should be used intraoperatively to confirm the diagnosis and evaluate surgical margins.

Since in this case melanonychia was accompanied by onycholysis and hyperkeratosis and associated with intraoperative confocal microscopy findings, the hypothesis of pigmented SCC *in situ* was raised and later confirmed by histopathologic examination.^8^

We therefore suggest that SCC should be included as a differential diagnosis of melanonychia striata, and that intraoperative dermatoscopy and confocal microscopy may help evaluate, possibly diagnose and even eventually guide removal of nail SCC.

## Conflicts of interest

None disclosed.
